# The Essential Physiology of Respiration

**Published:** 1879-09

**Authors:** J. R. Larzelere


### THE ESSENTIAL PHYSIOLOGY OF RESPIRATION.

By J. R. LARZELERE, M.D.

It is not my purpose in this paper, to present to the Academy the whole subject of respiration; or tax the minds of members with an account of all the physical agents which are ministerial to the accomplishment of this highly important function. All of you, doubtless, are acquainted with the anatomical construction of the lungs; in brief, their structures (emanating from without inwards) consist of an external serous, subserous areolar tissue, parenchyma, air cells, lobules, connective tissue, nerves, ganglia, lymphatics, blood vessels, bronchial tubes and glands, mucous membrane, epithelia, and ciliary bodies, etc.

Of these structures, which I desire most to direct your attention, are those of the epithelia and ciliary bodies, the cells of which are conical and squamous in form; the latter are mainly found in the alveoli; these forms of cells extend throughout the respiratory tree. Coupled with these living cells, are constantly present the nuclei, also, other characteristic peculiarities; of which the most important are the existence in some of them of ciliated prolongations, which latter have a continued vibratory movement; the rapidity of motion, at times, is estimated at from two hundred, or more, per second, and thus is perpetuated all through life.

It is a most interesting and remarkable life phenomenon, that these delicate structures should ever remain in constant motion, both during physiological and pathological conditions.

Epithelial cells having vibratile cilia, belong only to the higher order of animal organisms; they have never been observed to exist in the articula (insects.) These ciliary bodies are ever tending their motion in a uniform direction, that is, from the minute bronchial tubes onward through the larger bronchi to the mouth. The special function of the epithelial cilia would seem to consist in keeping the channels for the ingress and egress of air free from all matters which would become objects of bronchial obstruction. It is to the motion of these hair-like bodies, that dead epithelial scales, in the form of mucus, is removed from the alveoli and bronchi. The recovery of many of our pneumonic and phthisical patients is largely attributable to the action of these little bodies. So far as known, the nervous system has nothing to do with keeping up their incessant vibratory motions.

The life phenomena of the cilia are so intimately connected with the cells that if they should be detached they immediately cease to move; thus demonstrating the paternity of the cell, especially of the protoplasmic element within the same.

The vibratile movements of these epithelial cilia are checked by anæsthetics, (ether, chloroform, etc). The absence of oxygen appears to paralyze them, and induce asphyxia.

Acids retard, while alkalies accelerate their motion, also, a low temperature abates, whilst a high one increases their activity.

It is seemingly evident that the initial respiratory change takes place at the epithelium of the minute bronchi, as well as that of the alveoli. The epithelium of the bronchial mucous membrane readily allows the production of hematosis (or gaseous exchanges). That this is true, you need not be reminded of the anatomical fact, that the bronchial arteries have no corresponding veins, and it is through their blood that the bronchi receives nourishment, is oxidized by contact with this air, and flows immediately into the pulmonary veins, along with the arterial blood and is carried back the left heart.

Nutrition and oxidation take place in the lung from the contact of air with the blood furnished by the bronchial arteries. The most marked function of the epithelia at their free surfaces is to preside over the interchanges of nutrition; and especially is this true of the pulmonary epithelium, which is obviously ministerial to the so-called absorbtion of the fluid and gaseous materials. The pulmonary epithelium verily may be regarded as the primordial structure, which aids in the first step of the complex function of respiration.

Recall to your minds but for a moment some pathological conditions which will aid you in perceiving the great importance of the epithelium, not only on the lungs but including all the general organs of which their structure is partially composed, viz.: so-called pseudo-membranous inflammation of the respiratory tract consists principally of hypertrophy of the tracheal and bronchial epithelium; also, in the deep seated epithelium of the pulmonary vesicles, the condition denominated pnemonia, is, mainly, a change of the epithelium of the vesicles ; and it is to the filling of the latter with dead fragments of the epithelium, which excludes the air from the alveoli, developing that condition in pathology known as hepatization. Furthermore, so-called tubercle is first hypertrophy, and subsequently mummified deposits incubated from dessicated vesicular epithelium.

It is only of comparatively recent date that physiologists have deigned to attach much importance to the epidermis, having regarded it as a kind of secretory product of the dermis, yet, in verity, it is this structure which is most often affected in diseases of the skin. The maximum number of affections called dermatoses are only epidermatoses, a pathalogical state of the cutaneous epithelium. In like manner, may we look upon acute and chronic effusions of serous structures, when not produced by mechanical causes, as generally referable to a partial or total loss of function of their epitheliums.

Hence we may conclude with certainty that the normal functions of the epithelial globule is to choose select materials, draw unto themselves certain elements from the surrounding mediums; also, reject and give egress to others.

In the lungs, it is the function of the epithelial globule to imbibe, take hold upon, seize on the oxygen of the air, and retain it (so to speak,) in readiness for the reduced oxyhæmoglobin which is coursing the pulmonary arteries to reach the capillaries, at which point the retained oxygen is taken up by the red globules.

To make use of a simile, the pulmonary epithelium may be likened to a faithful sentinel, who with increasing vigilance guards the post of duty, the better to prevent the ingress or egress of factors, whose presence would prove detrimental to the cause over which he has control.

Normal respirable air is composed of definite parts of oxygen and definite parts of nitrogen, with a mere shadow of carbonic acid. It is the first named gas which is so essential to the metabolisms of the living economy, the inspired nitrogen so far as known undergoes no marked mutation, and is expired in about the same quantity as it entered the lungs.

That the large per centage of nitrogen which enters into the composition of the air, and yet, when taken into the economy, should there perform no important function, to us, is most truly remarkable. On the other hand, may we not, with some plausible show of presumption, regard it as one of those occult forces, which, when taken into the organism, is utilized as a balance wheel to other forces of the body.

As before intimated, the blood as it courses through the pulmonary capillaries undergoes important changes; the extravascular structure which first aids in this modification is the pulmonary epithelia, the normal function of which is to hold in store the gases imbibed or absorbed by the reduced hæmoglobin, whence the vascular current is known as arterial blood on its way through the pulmonary veins to the left side of the heart, hence throughout the arterial system to all the tissues of the body. The differential character of arterial and venous blood consists in the relative proportion of the oxygen and carbonic acid gases contained in each. Some of the older teachers affirmed that the function of the red globule was to dissolve as well as to transport the oxygen.

It is now generally taught that the globules are simply carriers of oxygen, in which state they are known as oxyhæmoglobinized, and it is to this combination which imparts to them their bright scarlet color. Of the whole quantity of oxygen in the blood, only a small part is absorbed, in obedience to the law of pressures (the so-called Henry Dalton law.) The large per centage is in a state of combination with the hæmoglobin which readily combines with the oxygen of the air to which it is exposed, so also, does dissociation readily occur at low pressures, or in the presence of indifferent gases, or by the action of substances having a greater affinity for oxygen than hæmaglobin. These mutations occur in those structures of the body where combustion takes place; the extra-vascular elements having a super-affinity for oxygen, over and above that of the hœmoglobin. And just here, at the expansive capillary area, is where the red globule gives up its claim to the oxygen, and it is passed over to the immediate extra-vascular tissues or structures, and what further changes occur at this point, is now what is most perplexing the brain of the physiologist to explain.

It is generally conceded that at or about this topography of the body, assimilation and disassimilation occurs, and he who possesses the wisdom to first explain this hitherto inexplicable function, will obtain a posthumous reputation not excelled by any of the great lights of the past.

As the result of the presence of oxygen at the expansive vascular area of the body, there is induced the genesis of carbonic acid, and the same interchange of gases occur here as at the lungs, save only that the order of exchange is inversed, the globule parting with its oxygen, while the carbonic acid seeks egress in the liquor of the blood, and from thence is conveyed to the lungs for expulsion. However obscure may be these phenomena to our mind, they are naught but tissue respiration; the tissues do breathe, the only difference being the inversed order of the gaseous exchanges and the location of the tissues. In the lungs, the oxygen can only be utilized when combined intra-vascular, at the great vascular area, the extra-vascular tissues take charge of and associate with the oxygen, after the wisdom of "Him who guides the wandering star through trackless realms of ether's space."

As before intimated, just what metabolism of the cell, or its immediate protoplastic surroundings, is induced by the interchange of the gases, is not at all satisfactory to the physiologist.

Nevertheless, I entertain a feeling of professional pride for the zeal which has been so often put forth on this asbtruse point in physiology by two able members of the Academy. Although their teachings on the subjects to us juniors seem diametrical, yet do I admire their temerity. One of them maintains that the cell in the performance of normal function is the object of destructive metamorphosis, while the other, with equal tenacity, affirms that such change or changes are confined to the intra and extra cell surroundings, and that the cell itself "goes scott free."

Beholding this great question from our own personal, unenlighted stand-point, it would verily seem as though some modification of structural or tissue element or elements must occur from the inter-action of the exchange of the forces; whether such metabolism is at the expense of structure, no longer able to perform normal function, or, on the other hand, whether the gaseous forces are utilized by the elementary tissues on their way to the development of structure, or both, is as yet the undiscovered palm star in physiology, and still remains an inviting field, for the former alluded to learned representatives of the profession to spend much time in research and thought.

As before stated, respiration takes place not only in the lungs, but in the most intimate portions of the tissues. They themselves do actually breathe, in a chemical sense; thus, if a muscle be detached from the body soon after death, and suspended in an oxygenated atmosphere, it will consume oxygen and evolve carbonic acid; this combustion is intensified if the muscle is made to contract. The breathing capacity of the red globule for oxygen depends upon its perfect condition. Oxygen is truly food for the blood globule, a part of which it assimilates for its own preservation, as also do the muscle and nerve cells; the latter consume largely of oxygen when under strong and continued labor, individuals of studious habits require a greater amount of oxygen on which the nerve cells may feed.

Compounds of antimony and arsenic, phosphorus, and the salts of soda, and the acids of the bile, are agents which materially change the shape of the globule, and greatly interfere with the performance of its physiological function, as evidenced in the urine, which is found to contain abnormal principles, such as the coloring matters of the bile and albumen.

The researches of Manassein seem to demonstrate the fact that the dimensions of the red globules are least, when, from a pathological increase of activity, they are in a condition to give up a larger percentage of oxygen, viz., (as in fever) when placed in any condition which increases the difficulty of absorption, (as when under the influence of carbonic acid and morphia). Under other surroundings they increase in size whenever they are brought in contact with any medium which contains a larger amount of oxygen, or, are placed under circumstances which tend to check the loss of oxygen, as when under the influence of refrigerents, oninia, alcohol, hydrocyanic acid, etc.

The lungs are regarded as the important organs of respiration, but from the prospective light which seems to exist in the near future, may cause us to look upon them as only performing or representing a link in the long chain of respiratory processes, which may commence in the very depths of the histological elements, and terminate in those surfaces which come in contact with the external medium.

The mechanical phenomena of respiration do not by any means constitute the initial processes of respiration, but simply are the result of a force evolved from the respiratory centre found in the medulla oblongata (bulb) which is transmitted by way of the centrifugal conductors, followed by reciprocal transmissions of afferent impulses, through the centripetal tracts back to medullary bulbs. This great moving centre not only receives and is acted upon by centripetal influences, but is also automatic. This central localle of dynamics is greatly affected by the condition of the blood, the less arterial the latter, the greater is the activity of the respiratory centre. In any obstruction or hindrance to the ingress of air into the lungs, or if the respiratory activity of the tissues are increased, as during active muscular labor, the blood becomes less arterial, more accumulation of carbonic acid, *pari passu* does the respiratory centre become vehement, and its force is eliminated through every available tract, until most of the muscles of the body are made to feel its effects. A marked illustration of a surplus increase of these gases in the blood is attended by diametrical results, as evidenced in the conditions known as apnœa and dyspnœa; the former is induced by the presence of surplus oxygen, while the latter is caused by the increased amount of carbonic acid, which brings us to the conclusion that the activity of the respiratory centre is dependent on the conditions of the blood; being quickened when the blood is more venous, and depressed when supplied with surplus oxygen.

That the co-ordinated impulses do descend from the medulla along the efferent nerves, is proved by the fact that the removal or injury of the medulla alone at once stops all respiratory movements, even if a small portion of the medulla, especially that portion which lies below the vaso-motor centre, immediately intervening between which and the calamus scriptorius, is in the least injured, respiration ceases for all time, though any other part of the body be left intact.

The respiratory centre is greatly influenced by extrinsic agents, such as volition, emotion, cold water, and all other outward contact which induces epidermic excitations. However much the character of the centripetal impulses may affect the respiratory centre, it is evident that the centre is endowed with the function of starting efferent impulses de novo.—*Cincinnati Lancet and Clinic.*

